# Clinical Outcome of COVID-19 Patients Presenting With Gastrointestinal Symptoms

**DOI:** 10.7759/cureus.15710

**Published:** 2021-06-17

**Authors:** Batool Abro, Jamil M Bhatti, Ali Akbar Siddiqui

**Affiliations:** 1 Internal Medicine, Shaheed Mohtarma Benazir Bhutto Medical University, Larkana, PAK; 2 Infectious Diseases, The Cancer Foundation Hospital, Karachi, PAK; 3 Infectious Diseases, Dr. Ziauddin Hospital, Karachi, PAK; 4 Gastroenterology and Hepatology, Isra University Hospital, Hyderabad, PAK

**Keywords:** covid-19, gastrointestinal symptoms, mechanical ventilation, non-invasive ventilation, intensive care unit

## Abstract

Background

Patients with coronavirus disease 2019 (COVID-19) usually have fever and respiratory tract complaints; however, many report gastrointestinal (GI) symptoms. The frequency of GI symptoms ranges from 16% to 61%. Although COVID-19 morbidity and mortality are related to pulmonary disease, poor outcomes have been linked to GI symptoms. Therefore, this study aimed to determine the outcomes of COVID-19 patients who presented with GI symptoms.

Methods

We conducted a retrospective cohort study at Isra University Hospital in Hyderabad, Pakistan, from April 2020 to October 2020.

Results

In total, 395 polymerase chain reaction-positive individuals were included. No differences in age or comorbidities were found. Of the 84 patients who needed intensive care unit admission, 17 had GI symptoms (P= 0.357). Moreover, GI symptoms were reported in 9/42 patients who required mechanical ventilation (P = 0.674) and 35/184 patients who required non-invasive ventilation (P = 0.029). GI symptoms were reported in 47/206 patients discharged on room air (without supplemental oxygen) (P= 0.549), 11/77 who died (P = 0.025), 2/11 who were referred elsewhere due to financial issues (P = 0.999), 7/32 who left against medical advice (P = 0.764), and 28/69 who were discharged requiring oxygen at home (P* = *0.001).

Conclusions

Patients with GI symptoms had reduced odds of mortality, and increased odds of discharge requiring supplemental oxygen.

## Introduction

The novel coronavirus identified in late 2019 was named the severe acute respiratory syndrome coronavirus 2 because of its structural similarity with the severe acute respiratory syndrome virus. In February 2020, its resulting disease was established as coronavirus disease 2019 (COVID-19) [1]. Common presentations include fever and symptoms related to the respiratory tract; however, many COVID-19 patients report gastrointestinal (GI) symptoms. Different frequencies of GI symptoms have been reported worldwide, ranging from 16% to 61% [2,3]. Pulmonary disease and thromboembolic phenomena are major causes of mortality and morbidity in patients with COVID-19, and studies have reported that some patients may present with GI symptoms, which may precede the development of pulmonary symptoms [3,4]. To date, no study has been conducted on the outcomes of COVID-19 in patients with GI symptoms on presentation in Pakistan.

## Materials and methods

We conducted a retrospective cohort study at a tertiary care hospital in Pakistan from April 2020 to October 2020. The study included all polymerase chain reaction (PCR)-positive COVID-19 patients tested through a nasopharyngeal swab by reverse-transcription assay. Patients with two to three or more loose stools or vomiting, anorexia, and nausea within three to five days of testing were considered to have primary symptoms of interest. Death, discharge on room air (without oxygen [O_2_]), and discharged requiring O_2_ were considered outcomes of primary interest, while the need for intensive care unit (ICU) admission, non-invasive ventilation, and assisted mechanical ventilation were outcomes of secondary interest. Other variables included age, gender, comorbidities, radiological findings, laboratory parameters, and other common symptoms of COVID-19. Patients who had a history of GI diseases or took any medication for GI symptoms were excluded. Institutional Review Board approval was obtained before conducting the study. We categorized discharged patients as those discharged on room air (without supplemental oxygen) and those who improved and were discharged anytime during their hospital stay requiring supplemental oxygen at home. Furthermore, those who could not stay due to financial issues were referred to other hospitals, and those who left against medical advice (LAMA) were labeled as such.

 

Data collection

We first collected a complete list of patients hospitalized with a diagnosis of PCR-positive COVID-19 through their electronic records. Demographics, clinical features, radiological findings, comorbidities, outcomes, and laboratory parameters were extracted through a detailed chart review. We also manually reviewed the patients’ charts for chief complaints and history of presenting illness to ensure accurate data capture. A total of 415 charts were reviewed, of which three were excluded because of a concomitant diagnosis of inflammatory bowel disease, two were excluded because of a concomitant diagnosis of irritable bowel syndrome, and 15 were excluded because of incomplete medical records.

Statistical analysis

We entered and analyzed the data using SPSS version 24 (IBM Corp, Armonk, NY, USA). We computed the median and interquartile range for continuous variables instead of mean and standard deviation; as data were not evenly distributed, when analysis was done, there was violation of the assumption of normality. Frequencies and percentages were computed for categorical variables, and their proportional differences were compared using the chi-squared test. Outcomes were computed using univariate and multivariate analyses. Kaplan-Meier analysis was performed to analyze patient survival. Statistical significance was set at P <0.05.

## Results

A total of 395 PCR-positive individuals were included. Hospital courses and demographic, clinical, and radiological characteristics are shown in Table 1.

**Table 1:** Demographic characteristics, features, radiologic findings, and hospital course of patients on presentation CKD, chronic kidney disease; ESRD, end-stage renal disease; NIV, non-invasive ventilation; ICU, intensive care unit. *Fisher's exact test.

	Total	GI Symptoms n = 95	No GI Symptoms n = 300	P-Value
Age	395	55.4 ± 13.7	55.8 ± 14.7	0.838
Gender				
Male	266	71 (74.7%)	195 (65.0%)	0.078
Female	129	24 (25.3%)	105 (35.0%)	
Comorbidities				
Diabetes mellitus	194 (49.1%)	49 (51.6%)	145 (48.3%)	0.581
Hypertension	190 (48.1%)	49 (51.6%)	141 (47.0%)	0.436
CKD/ESRD	12 (3.0%)	2 (2.1%)	10 (3.3%)	0.543
Asthma	28 (7.1%)	9 (9.5%)	19 (6.4%)	0.299
Ischemic heart disease	58 (14.7%)	16 (16.8%)	42 (14%)	0.495
Chronic liver disease	7 (1.8%)	3 (3.2%)	4 (1.3%)	0.366*
Malignancy	10 (2.5%)	2 (2.1%)	8 (2.7%)	0.999*
Smoking	45 (11.4%)	11 (11.6%)	34 (11.3%)	0.948
Symptoms				
Fever	352 (89.1%)	88 (92.6%)	264 (88.0%)	0.207
Cough	300 (75.9%)	62 (65.3%)	238 (79.3%)	0.005
Shortness of breath	309 (78.2%)	63 (66.3%)	246 (82.0%)	<0.001
Lethargy	154 (38.9%)	65 (68.4%)	89 (29.7%)	<0.001
Myalgia	93 (23.5%)	35 (36.8%)	58 (19.3%)	0.001
Headache	57 (14.4%)	20 (21.1%)	58 (19.3%)	0.714
Anosmia	97 (24.6%)	28 (29.5%)	69 (23.0%)	0.201
Runny nose	47 (11.9%)	18 (18.9%)	29 (9.7%)	0.015
Hospital Course				
ICU admission	84 (21.3%)	17 (17.9%)	67 (22.3%)	0.357
NIV	184 (46.6%)	35 (36.8%)	149 (49.7%)	0.029
Mechanical ventilation	42 (10.6%)	9 (9.5%)	33 (11.0%)	0.674
Radiological Findings				
Bilateral infiltrates	272 (68.9%)	58 (61.1%)	214 (71.3%)	0.059
Peripheral infiltrates	277 (70.1%)	65 (68.4%)	212 (70.7%)	0.677
Ground glass opacities	96 (24.3%)	30 (31.6%)	66 (22.0%)	0.058
Consolidation	105 (26.6%)	23 (24.2%)	82 (27.3%)	0.548
Right lung involvement	322 (81.5%)	73 (76.8%)	249 (83.0%)	0.178
Left lung involvement	286 (72.4%)	60 (63.2%)	226 (75.3%)	0.021

There were 95 patients with GI symptoms and 300 without GI symptoms. The mean ages of individuals with and without GI symptoms were similar (55.43 vs. 55.78 years). Two hundred sixty-six patients were male, and 129 were female. Diabetes mellitus was the most common comorbidity and was found in 194 patients, followed by hypertension in 190 patients. Fever was the most common symptom and was found in 352 patients, of whom 88 also had GI symptoms. Shortness of breath and cough were found in 309 and 300 patients, of whom 63 and 62 had GI symptoms, respectively (P = 0.001; P = 0.005). Other common symptoms included lethargy, anosmia, and myalgia. Of the GI symptoms, anorexia was the most common presenting symptom, followed by diarrhea, nausea, and vomiting (Table 2).

**Table 2:** GI symptoms of patients with coronavirus disease 2019 on presentation GI, gastrointestinal.

GI Symptoms	Frequency
Anorexia	75 (19%)
Nausea	59 (14.9%)
Vomiting	40 (10.1%)
Diarrhea	70 (17.7%)

Peripheral infiltrates were the most common radiological finding, with the right lung most commonly affected in our patient population. Other radiological findings included bilateral infiltrates, ground-glass opacities, and consolidation. Of the 84 patients who required ICU admission, 17 had GI symptoms (P = 0.357); of the 42 who required mechanical ventilation, nine had GI symptoms (P = 0.674); and of the 184 who were put on non-invasive ventilation, 35 had GI symptoms (P = 0.029). Baseline laboratory parameters are shown in Table 3.

**Table 3:** Baseline laboratory parameters GI, gastrointestinal; ALT, alanine transaminase; AST, aspartate transaminase; LDH, lactate dehydrogenase, IQR, interquartile range; FEU, fibrinogen-equivalent units.

	GI Symptoms Median (IQR) n = 95	No GI Symptoms Median (IQR) n = 300	P-Value
White blood cells (×10^9^/L)	9.0 (7.0-13.0)	11.0 (8.0-17.0)	0.005
Hemoglobin (g/dL)	12 (11-13)	12 (11-13)	0.939
Lymphocytes (%)	15 (7-25)	10 (5-19)	0.005
Platelets (×10^9^/L)	273 (177-343)	238 (175-330)	0.337
ALT (U/L)	44 (25-70)	40 (27-65)	0.973
AST (U/L)	50 (34-78)	45 (31-65)	0.258
Creatinine (mg/dL)	1.0 (0.9-1.0)	1.0 (1.0-1.2)	0.002
D-dimer (ng/mL FEU)	1073 (527-2938)	1819 (773-7647)	0.005
LDH (U/L)	408 (296-562)	445 (320-653)	0.120
C-reactive protein	98 (39-189)	98 (39-189)	0.749
Serum ferritin (ng/mL)	915 (500-1451)	758 (356-1463)	0.158

On presentation, those with GI symptoms had a significantly lower median white cell count (9.0 × 10^9^ vs. 11 × 10^9^/L; P = 0.005), lower lymphopenia prevalence (15% vs. 10%; P = 0.005), and lower D-dimer (1073 vs. 1819 ng/mL; P = 0.005) than those without GI symptoms; lactate dehydrogenase (408 vs. 445 U/L; P = 0.120) and other inflammatory markers, such as serum ferritin, were not significantly different between groups. Patient outcomes are shown in Table 4.

**Table 4:** Outcome of patients GI, gastrointestinal; RA, room air; LAMA, left against medical advice; O_2_, oxygen. *Fisher's exact test.

Outcome	GI Symptoms n = 95	No GI Symptoms n = 300	P-Value
Discharged on RA	47 (49.5%)	159 (53.0%)	0.549
Expired	11 (11.6%)	66 (22.0%)	0.025
Referred	2 (2.1%)	9 (3.0%)	0.999*
LAMA	7 (7.4%)	25 (8.3%)	0.765
Discharged on O_2_	28 (29.5%)	41 (13.7%)	0.001

GI symptoms were reported in 47/206 patients ultimately discharged without requiring O_2_ at home (P = 0.549), 11/77 who died (P = 0.025), 2/11 who were referred to another facility due to financial issues (P = 0.999), 7/32 who LAMA (P = 0.765), and 28/69 who were discharged requiring O_2_ at home (P = 0.001). On multivariable analysis (Table 5), patients who had GI symptoms on presentation had lower odds of death (odds ratio [OR], 0.464; P = 0.035), lower odds of requiring non-invasive ventilation (OR, 0.591; P = 0.029), and higher odds of requiring at-home O_2_ therapy (OR, 2.640; P < 0.001). Furthermore, individuals with GI symptoms had lower odds of requiring invasive mechanical ventilation (OR, 0.847), requiring ICU admission (OR, 0.758), and having a severe disease (OR, 0.854) than individuals without GI symptoms, but these differences were not statistically significant (P = 0.675; P = 0.358; P = 0.604, respectively).

**Table 5:** Multivariate analysis of GI symptoms CI, confidence interval; IMV, invasive mechanical ventilation; NIV, non-invasive ventilation; ICU, intensive care unit; RA, room air; O_2_, oxygen.

	Odds Ratio	95% CI	P-Value
Gender	0.628	0.373-1.056	0.078
Expired	0.464	0.234-0.921	0.035
Discharged on RA	0.868	0.547-1.378	0.550
Discharged on O_2_	2.640	1.522-4.578	<0.001
IMV	0.847	0.390-1.840	0.675
NIV	0.591	0.368-0.950	0.029
ICU admission	0.758	0.420-1.368	0.358
Severe disease	0.854	0.471-1.549	0.604
Critical disease	0.527	0.214-1.298	0.159

## Discussion

COVID-19 has posed extraordinary challenges to global healthcare systems. The majority of patients present with fever, lethargy, or pulmonary manifestations; however, almost all organs of the body can be involved, and GI symptoms are among those commonly reported [5]. Moreover, viral shedding in the feces of patients has been reported in the literature worldwide [6]. In early 2020, many patients with GI symptoms were overlooked by clinicians, resulting in the transmission of infection to other patients and increased morbidity and mortality as a result of late diagnosis [3].

Here, we found no differences in mean age between the two groups. There was also no significant difference in comorbidities, which contradicts the findings of Laszkowska et al. [7]. This study concluded that 24.1% of patients included here had GI symptoms. This prevalence was lower than that reported by some studies from China and the USA, which described figures ranging from 50.5% to 61.3% [8,9]. However, this prevalence was higher than the 10.3% prevalence reported by a study in India [10], as well as the 17.6% prevalence reported by a meta-analysis of >59 studies and >4000 patients [11]. A lower prevalence of GI symptoms has also been observed in the USA in a recent meta-analysis of >10,000 patients and another of >8000 patients; these studies reported a prevalence of 7.8% [12] and 10% [13], respectively. Anorexia was found to be the most common GI symptom in our study; when we excluded anorexia, only 19% of patients had GI symptoms, as some identified anorexia as a non-specific complaint found in many other conditions. This study reported diarrhea in 17.7% of patients, nausea in 14.9%, and vomiting in 10.1%; this was inconsistent with findings of one American study that reported diarrhea and nausea in 33% and 26.4% of patients, respectively [2], as well as with those from a meta-analysis that reported diarrhea in 7.4% of patients and nausea or vomiting in 4.6% [14].

This study found significant differences in the outcomes of patients with GI symptoms on presentation. A significant association was found between the presentation of GI symptoms and lower mortality; patients with GI symptoms had lower mortality rates than those without. Furthermore, patients were more likely to be discharged requiring supplemental O_2_ if they had GI symptoms on presentation. This finding is not consistent with the findings of Ramchandran et al. and Redd et al., who found no association between GI symptoms and worse clinical outcomes of COVID-19 [2,15]. However, Pan et al. concluded that COVID-19 patients with GI symptoms had lower recovery and discharge rates than those without GI symptoms [16]. Other studies have reported significant associations between poor outcomes, more severe illness, and GI symptoms [4,8]. Significant associations have been found in other studies [17,18]. This study’s findings are consistent with those of Laszkowska et al., who found better outcomes in COVID-19 patients with GI symptoms, including lower rates of mortality and lower rates of requiring mechanical ventilation [7]. 

This study reports that COVID-19 patients with GI symptoms had significantly lower D-dimer levels than those without such symptoms, while serum ferritin, lactate dehydrogenase, and C-reactive protein levels were not significantly different. In contrast, Laszkowska et al. demonstrated lower C-reactive protein and other markers, including D-dimer and lactate dehydrogenase levels [7]. In one multicenter study, Zhou et al. also found elevated C-reactive protein levels in COVID-19 patients with GI symptoms [19]. Moreover, this study found a significant difference in lymphocyte count between the two groups; COVID-19 patients with GI symptoms had a higher median lymphocyte count than those without GI symptoms. In contrast, Chen et al. reported a negative relationship between circulating lymphocyte count and initial GI symptoms [20].

One important strength of our study is the validation of our results from other studies by Laszkowska et al. [7]. Additionally, to the best of our knowledge, this is the first study from Pakistan to evaluate outcomes of patients with GI symptoms on presentation.

This study has a few limitations, including its retrospective design, that it was a single-center study with a small sample size, and its reliance on medical records rather than direct patient histories. This could result in selection bias and limit the generalizability of the results. Another limitation of this study was the inclusion of only hospitalized patients, which does not reflect the true prevalence and outcome of COVID-19 patients with GI symptoms.

## Conclusions

This study concluded that COVID-19 patients with GI symptoms on presentation had decreased odds of mortality, and increased odds of discharge requiring supplemental oxygen. Inflammatory markers were lower in patients with GI symptoms, which may be a possible cause of indolent disease in these patients. Further data are required to confirm this association.
